# Elevation of the unfolded protein response increases RANKL expression

**DOI:** 10.1096/fba.2019-00032

**Published:** 2020-01-31

**Authors:** Srividhya Iyer, Christian Melendez‐Suchi, Li Han, Giulia Baldini, Maria Almeida, Robert L. Jilka

**Affiliations:** ^1^ Department of Orthopaedic Surgery University of Arkansas Medical Sciences Little Rock AR USA; ^2^ Division of Endocrinology and Metabolism Center for Osteoporosis and Metabolic Bone Diseases University of Arkansas Medical Sciences Little Rock AR USA; ^3^ Department of Biochemistry and Molecular Biology University of Arkansas Medical Sciences Little Rock AR USA; ^4^ Central Arkansas Veterans Healthcare System Little Rock AR USA

**Keywords:** calvaria, cytokines, endoplasmic reticulum stress, osteoblast, osteocyte

## Abstract

Increased production of the osteoclastogenic cytokine RANKL is a common feature of pathologic bone loss, but the underlying cause of this increase is poorly understood. The unfolded protein response (UPR) is activated in response to accumulation of misfolded proteins in the endoplasmic reticulum (ER). Failure to resolve misfolding results in excess UPR signaling that stimulates cytokine production and cell death. We therefore investigated whether RANKL is one of the cytokines stimulated in response to elevated UPR in bone cells. Pharmacologic induction of UPR with tunicamycin (Tm)‐stimulated RANKL expression in cultures of primary osteoblastic cells and in osteoblast and osteocyte cell lines. Pharmacologic inhibition of the UPR blunted Tm‐induced RANKL production. Silencing Edem1 or Sel1l, proteins that aid in degradation of misfolded proteins, also induced UPR and increased RANKL mRNA. Moreover, Tm or hypoxia increased RANKL and bone resorption in cultures of neonatal murine calvaria. Administration of Tm to adult mice caused dilation of ER in osteoblasts and osteocytes, elevated the UPR, and increased RANKL expression and osteoclast number. These findings support the hypothesis that excessive UPR signaling stimulates the expression of RANKL by osteoblasts and osteocytes, and thereby facilitates excessive bone resorption and bone loss in pathologic conditions.

**Abbreviations**
ATF6: activating transcription factor 6
CHOP: C/EBP homologous protein 10
ER: endoplasmic reticulum
ERAD: ER‐associated degradation
IL: interleukin
Ire1α: Inositol‐requiring enzyme1α
Ob: osteoblast
Ot: osteocyte
PERK: PKR‐like ER kinase
RANKL: receptor activator of NFκB ligand
sXBP1: spliced X‐box binding protein
Tm: tunicamycin
TNF: tumor necrosis factor
UPR: unfolded protein response
VEGF: vascular endothelial growth factor

## 1 INTRODUCTION

Osteoclast formation and activity strictly depends on the cytokine receptor activator of NFκB ligand (RANKL).^1^ Osteocytes embedded within the bone matrix are a critical source of RANKL, the expression of which is controlled by the parathyroid hormone under physiologic conditions.^2^, ^3^ Studies in mice have shown that excessive production of RANKL by osteocytes in hyperparathyroidism leads to increased osteoclastogenesis, bone resorption, and bone loss.^4^ High levels of RANKL are also associated with and in some cases causally linked with other bone diseases like arthritis, orthopedic implant‐associated osteolysis, periodontitis, age‐dependent osteoporosis, as well as postmenopausal osteoporosis, and unloading‐induced bone loss.^5^, ^6^, ^7^, ^8^, ^9^, ^10^, ^11^, ^12^, ^13^ Some of these conditions are associated with increased inflammatory cytokines such as interleukin (IL)‐1 and tumor necrosis factor‐α (TNF‐α), that can directly stimulate RANKL synthesis and thereby augment bone resportion.^14^, ^15^ In general, however, the signaling pathways and molecular mechanisms that contribute to elevated cytokine production in pathologic bone loss are not well defined.

Accumulation of protein damage and/or misfolding increases with age and contributes to progression of multiple age‐related diseases.^16^, ^17^, ^18^ The folding of transmembrane and secreted proteins occurs in the endoplasmic reticulum (ER). This process is mediated by chaperones and enzymes that catalyzes glycosylation, prolyl isomerization, and the formation of disulfide bonds.^19^, ^20^ Changes in nutrient supply, redox status, intracellular calcium, or secretory demand can increase the level of misfolded proteins, which are then cleared by the ER‐associated degradation (ERAD) system. Accumulation of misfolded proteins results in ER stress leading to activation of the unfolded protein response (UPR) that restores proteostasis by stimulating ERAD and increasing protein folding capacity.^21^, ^22^ Chronic or unresolved ER stress initiates proapoptotic signaling as well as increased production of inflammatory cytokines like TNF‐α, IL ‐6, interferon‐γ, and other factors such as vascular endothelial growth factor (VEGF), that are involved in tissue repair.^23^, ^24^, ^25^

The UPR is indispensable for the optimal function of secretory cells. For example, loss of function mutations in PERK, one of the sensors of ER stress, underlie the multiple abnormalities seen in patients with Wolcott‐Rallison syndrome, including short stature, low bone mass, neuromotor defects, hepatic and renal failure secondary to early‐onset diabetes mellitus.^26^ Skeletal disease often involves alterations in redox status, hypoxia, and other factors that could alter protein folding.^27^, ^28^ These conditions could induce ER stress and the UPR in bone cells, particularly matrix synthesizing osteoblasts, and osteocytes, which secrete a variety of factors, involved in control of bone formation and resorption.^29^, ^30^ Therefore, we sought to determine whether UPR can stimulate production of RANKL and perhaps other pro‐resoprtive cytokines by bone cells. We report that this is indeed the case, and that this response is associated with increased osteoclast number and bone resorption.

## 2 MATERIALS AND METHODS

### 2.1 Animals

All animal procedures were approved by the Institutional Animal Care and Use Committees of the University of Arkansas for Medical Sciences, and the Central Arkansas Veterans Healthcare System. Ten‐week‐old male C57BL/6J mice were injected intraperitoneally with 5 μg/g body weight tunicamycin (Tm) (T7765, Sigma‐Aldrich, St. Louis, MO, USA) and euthanized after 6, 24, or 48 hours. Tm was dissolved in 150 mmol/L glucose containing 2% DMSO (Sigma‐Aldrich). Mice injected 150 mmol/L glucose containing 2% DMSO were euthanized immediately and served as controls (vehicle). For qPCR analyses of cortical bone, the proximal and distal ends of femur and tibia were removed, the bone shafts flushed with PBS to remove marrow and scraped on the outside to remove adherent cells as described,^31^ and stored in liquid N2 until analysis. To assess osteoclast number, 4‐month‐old female C57BL/6J mice were injected with 0.3 μg/g body weight Tm or DMSO at 7 and 3 days before euthanasia. Tm doses and timeframes used for in vivo studies have been previously shown to induce UPR within a period that maximizes discovery of cytokine‐inducing properties without causing significant toxic effects that would negate or compromise our conclusions.^32^, ^33^

### 2.2 Cell cultures

Osteoblastic cells were isolated from neonatal calvaria as described before.^34^ Briefly, calvaria was dissected from 3 to 5‐day‐old pups (C57BL/6J background), trimmed in Hank's Balanced Salt Solution and washed with PBS containing 4 mmol/L EDTA. Cells were collected after sequential digestion with 200 U/mL collagenase type 2 (CLS2, Worthington Biochemical Corp.), each for 10 minutes, at 37°C were pooled and cultured in α‐MEM (GIBCO, Life Technologies) containing 10% preselected fetal bovine serum (FBS, HyClone, GE Healthcare, Chicago, IL, USA), 1% penicillin/streptomycin/glutamine and 50 µg/mL ascorbic acid. After expansion, cells were frozen in liquid nitrogen until further use. The bone marrow‐derived osteoblastic cell line UAMS‐32 (RRID:CVCL_D624)^35^ was maintained in α‐MEM containing 10% FBS and antibiotics. The osteocytic cell line MLO‐Y4 (RRID:CVCL_M098)^36^ was cultured on collagen‐coated plates in medium containing 2.5% fetal bovine serum and 2.5% bovine calf serum as described previously.^37^ For time course and gene expression studies, calvaria‐derived osteoblastic, UAMS‐32 or MLO‐Y4 cells were maintained in the presence of vehicle (0.1% DMSO) or Tm (in 0.1% DMSO). For studies with Perk and Ire1α inhibitors, calvaria‐derived osteoblastic or MLO‐Y4 cells were pretreated with 1 µmol/L GSK2606414 (CAS 1337531‐89‐1, EMD Millipore) or 100 µmol/L 4µ8C (CAS 14003‐96‐4, Calbiochem), respectively, for 1 hour, followed by addition of either vehicle (0.1% DMSO) or 2.2 µg/mL Tm (in 0.1% DMSO) for 4 hours. Protein concentration was measured using Bio‐Rad DC Protein Assay Kit (Biorad), as described previously.^37^

### 2.3 Organ cultures

Neonatal murine calvaria was dissected and cultured in DMEM (GIBCO, Life Technologies, Carlsbad, CA, USA) containing 15% preselected horse serum. On the following day, the organ cultures were transferred to fresh media. For gene expression studies, calvaria were treated with either vehicle or Tm for 4 hours, or cultured in media that was previously acclimatized to either 2% or 20% O_2_ for 6 hours. For bone resorption studies, the calvaria was transferred to a media‐containing vehicle or Tm for 4 hours, or to media acclimatized to 20% or 2% O2 for 6 hours. Thereafter, the bones were rinsed in fresh media and returned to the original culture media for 20 or 18 hours, respectively. The process was repeated daily for a total of 4 days. After the last treatment, the calvaria was frozen in liquid N2 for a later measurement of RNA, and the medium frozen for later measurement of Ca. (MAK022, Sigma‐Aldrich).^38^ Calcium release from calvaria was calculated by subtracting the Ca value determined in medium not exposed to calvaria from the value determined in medium from calvaria cultures obtained at the end of the experiment.

### 2.4 Silencing studies

Calvaria‐dervied osteoblastic cells were infected with lentivirus vectors expressing shRNAs for Edem1 or Sel1l (Mission RNAi; Sigma‐Aldrich) or control nontarget shRNA (SHC016V, Sigma‐Aldrich) transduction particles for 6 hours as previously described.^39^ The following lentivirus preparations were used for these experiments: TRCN0000018468 (edem1‐1), TRCN0000018469 (edem1‐2), TRCN0000250292 (Sel1l‐1), TRCN0000258035 (Sel1l‐2). Cells were then maintained in 1 µg/mL puromycin (Invitrogen, Life Technologies) for 10 days to select transduced cells. At approximately 80% confluence, cells were trypsinized and counted. There was no difference in cell number among the transduced cell preparations (not shown). Cells were then seeded as appropriate for subsequent qPCR analysis or immunostaining.

### 2.5 RNA isolation and TaqMan assay

Total RNA was extracted from cell cultures, calvaria or frozen bone shafts after homogenizing the samples in Trizol (Life Technologies) according to the manufacturer's instructions. The mRNA was reverse‐transcribed using the High‐Capacity cDNA Reverse Transcription Kit (Applied Biosystems). The cDNA was amplified by quantitative RT‐PCR using TaqMan Universal PCRMaster Mix (Life Technologies) according to the manufacturer's directions. The following TaqMan assays from Life Technologies were used: ATF4 Mm00515325_g1; CHOP Mm01135937_g1; sXBP1 (Forward 5′CTGAGTCCGCAGCAGGT3′, reverse 5′ TGTCAGAGTCCATGGGAAGA3′, probe FAM5′GGCCCAGTTGTCACCTCCCC3’NFQ); Edem1 Mm00551797_m1; Sel1l Mm01326442_m1; Herpud1 Mm00445600_m1; VEGF Mm00437306_m1; TNF Mm00443258_m1; RANKL Mm00441908_m1, and the house‐keeping gene ribosomal protein S2, Mm00475528_m1. Relative mRNA levels were calculated by normalizing to ribosomal protein S2 using the delta Ct method.^40^

### 2.6 Western blot analysis

Cells were washed twice with ice‐cold PBS and lysed with a buffer containing 20 mmol/L Tris‐HCL, 150 mmol/L NaCl, 1% Triton X‐100, protease inhibitor mixture, and phosphatase inhibitor cocktail (Sigma‐Aldrich). After incubation on ice for 30 minutes, the cell lysates were sonicated and centrifuged at 15 871 *g* for 15 minutes at 4°C. Protein concentration of cell lysates was determined using the Bio‐Rad DC Protein Assay kit (Biorad). Equivalent amounts of extracted protein (20‐40 μg per sample depending on the experiment) was subjected to 7%‐10% SDS‐PAGE gels and transferred electrophoretically onto polyvinyl difluoride membranes. The membranes were blocked in 5% fat‐free milk/Tris‐buffered saline for 90 minutes and incubated with each primary antibody followed by secondary antibodies conjugated with horseradish peroxidase. Monoclonal antibodies against p‐eIF2a (dilution 1:1000, #9721, Abcam, RRID:AB_330951), t‐eIF2a (dilution 1:1000, #9722, Abcam, RRID:AB_2230924), ATF6 (Dilution 1:500, NBP1‐40256, Novus Biologicals, RRID:AB_2058774), RANKL (1:1000 dilution, R&D systems, RRID: AB_2206198), and tubulin (dilution 1:5000, ab40742, Abcam, RRID:AB_880625) were used. The membranes were subjected to Western blot analysis with enhanced chemiluminescence reagents (Millipore). Quantification of the intensity of the bands in the autoradiograms was performed using a VersaDoc imaging system (Bio‐Rad).

### 2.7 Immunostaining

Protein retention by the ER was visualized by fluorescence microscopy using an antibody against the KDEL peptide, present in ER targeted proteins, as described previously.^41^, ^42^ Calvaria‐derived osteoblasts were cultured on collagen‐coated cover slips in 6‐well plates and fixed with 4% paraformaldehyde in PBS for 30 minutes at 4°C. Following permeabilization with solution containing 0.2% Triton X‐100, 100 µg/mL BSA, 0.01% sodium azide the cells were stained with anti‐KDEL antibody (1:200, ab12223, Abcam) in PBS containing 100 µg/mL BSA at room temperature for 1 hour. After 3 washes with 0.2% Triton X‐100, cells were incubated with Alexa Fluor®594 AffiniPure Goat Anti‐Mouse IgG (1:100, #115‐585‐003, Jackson Immunoresearch) for 1 hour and stained with DAPI. Images were captured as Z‐stacks with Zeiss LSM 880 Confocal Microscope using a 20X objective with constant parameters of acquisition (excitation wavelength: 405 and 561 nm). The z‐stacks were processed into a single 2D image using the Zen software. KDEL immunostaining was quantified using Image J software. First, a region of interest was selected by manually drawing the cell margin for each cell. Then, the average fluorescence pixel intensity of each cell in the red channel was determined.

### 2.8 Histology

To determine osteoclast number, femurs were fixed in 10% Millonig's formalin overnight, decalcified with 14% EDTA and embedded in paraffin. Five‐μm longitudinal sections were stained for TRAPase to visualize osteoclasts, and counter‐stained with toluidine blue. Histomorphometric measurements were done using the OsteoMeasure Analysis System (OsteoMetrics Inc) as previously described.^43^, ^44^ Analyses was restricted to the cancellous bone in the secondary spongiosa.

### 2.9 Electron microscopy

Marrow was flushed from the tibia after removing the epiphyses, and the bone fixed in 0.1 mol/L sodium cacodylate, pH7.4, containing 4% paraformaldehyde, 2.5% glutaraldehyde and 8.0 mmol/L CaCl_2_ at 4°C overnight, followed by decalcification with 14% EDTA for a week, as described previously.^45^ The shafts were trimmed to 1 mm length, postfixed with 1% osmium tetroxide, stained with 1% tannic acid and 0.5% uranyl acetate, and dehydrated in an ethanol series followed by propylene oxide. The samples were infiltrated and embedded in a mixture of Embed812 (Electron Microscopy Sciences), Araldite, dodecenylsuccinic anhydride, and DMP‐30. One hundred‐nm sections were cut with a DiATOME blade (Electron Microscopy Sciences) using an ultramicrotome (Leica Biosystems). The cross‐sections were adhered to copper grids (G100H‐Cu, Electron Microscopy Sciences) and examined at 80 kV using a transmission electron microscope (FEI Tecnai F20) equipped with a digital camera (FEI 4k Eagle).

### 2.10 Statistics

Data are shown as bar graphs with individual data points or dot plots. All values are reported as mean ± SD. Time course data in Figure 2 are plotted as mean ± SD. Statistical analyses were carried out using GraphPad Prism Version 7.04 (San Diego, CA). Data were analyzed using a one‐way ANOVA to detect statistically significant treatment effects, after determining that the data were normally distributed and exhibited equivalent variances. Multiple comparisons were evaluated with Dunnett's post hoc tests. *P*‐values less than .05 were considered significant. Data that did not pass the normality test after transformation were evaluated using the Kruskal‐Wallis Rank Sum Test.

## 3 RESULTS

### 3.1 Elevated UPR stimulates osteoclastogenic cytokine expression by cultured osteoblasts and osteocytes

**Figure 1:**
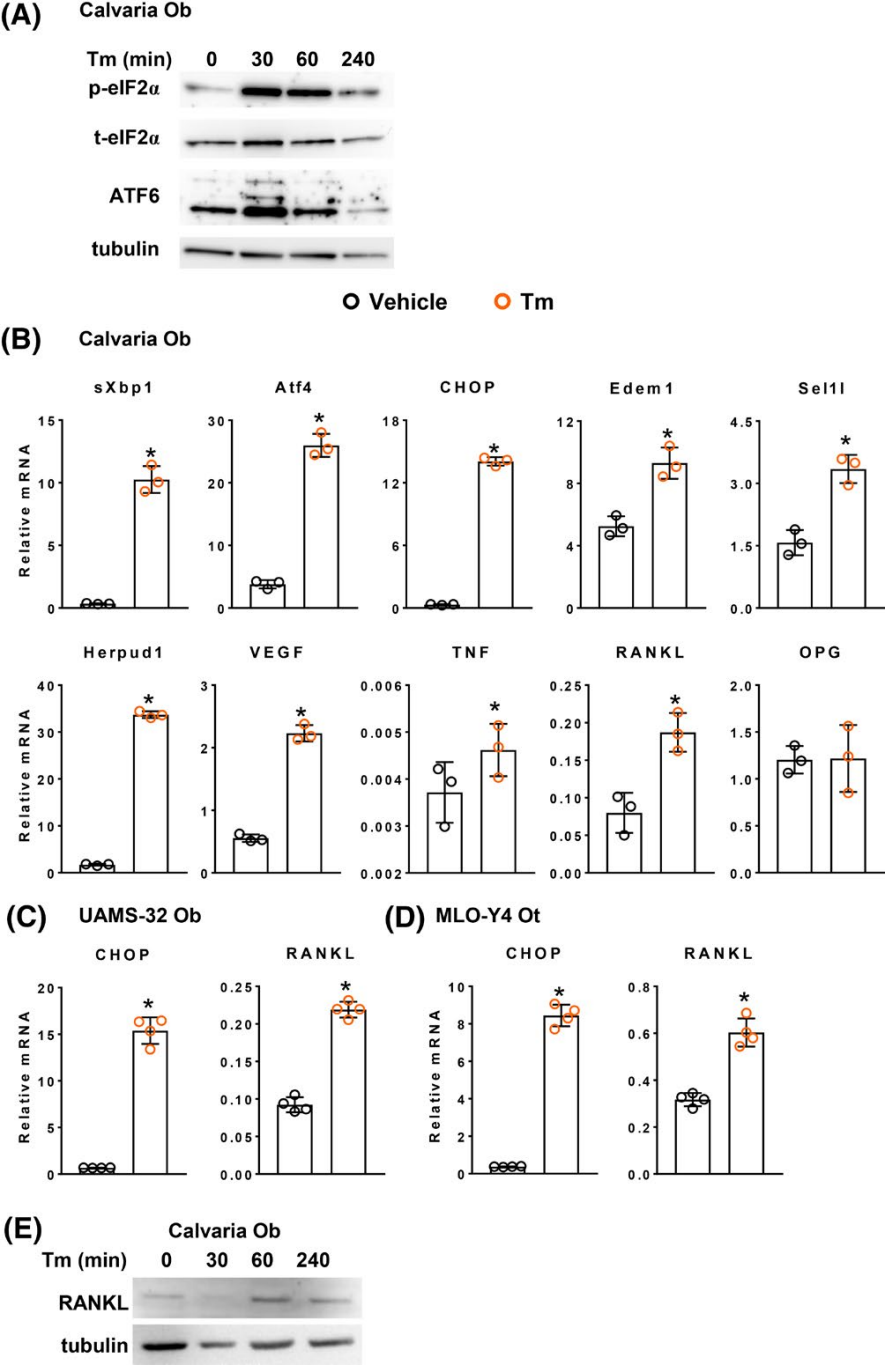
Tm‐induced UPR increases expression of RANKL in cultured osteoblastic and osteocytic cells. (A) Western blotting of cell lysates obtained from neonatal calvaria‐derived osteoblastic cells (Calvaria Ob) treated with 2.2 µg/mL Tm for the indicated times. (B‐D) Gene expression as determined by qRT‐PCR in (B) calvaria‐derived osteoblastic cells (n = 3/group) (C) Osteoblastic UAMS‐32 cells (UAMS‐32 Ob) (n = 4/group) or (D) osteocytic MLO‐Y4 cells (MLO‐Y4 Ot) (n = 4/group),maintained in presence of vehicle (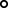, 0.1% DMSO) or 2.2 µg/ml Tm (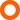) for 4 hours. (E) Western blot of RANKL protein in cell lysates obtained from calvaria‐derived osteoblastic cells as described in A in a separate study. Data shown are the mean and SD with individual data points. *P < .05 vs vehicle by Student's *t*‐test

Activation of the UPR is initiated by three ER transmembrane proteins namely, PERK (PKR‐like ER kinase), ATF6 (Activating transcription factor 6), and Ire1α (Inositol‐requiring enzyme1α).^22^, ^46^ In line with earlier studies in other cell types,^46^ addition of the N‐linked glycosylation inhibitor tunicamycin (Tm) – a commonly used pharmacologic tool for investigating the UPR – to calvaria‐derived osteoblastic cells increased phosphorylation of eIF2a, a target of PERK activity (Figure 1A). Administration of Tm concomitantly increased the levels of cleaved ATF6 protein indicating its activation. Downstream UPR signaling was also increased as measured by the transcript levels of sXBP1 (spliced X‐box binding protein) and ATF4, which are Ire1a and PERK targets, respectively (Figure 1B).^22^, ^46^ In addition, the mRNA abundance of the proapoptotic factor, CHOP (C/EBP homologous protein 10) was also increased. Tm also stimulated the expression of several components of the ERAD pathway including the mannosidase Edem1, as well as Sel1l, and Herpud1, which are components of the retro‐translocation complex in the ER membrane. Importantly, Tm‐induced UPR was associated with increased expression of VEGF, TNF, and RANKL, but not osteoprotegerin (OPG), the soluble decoy receptor for RANKL.^47^ Tm also increased RANKL expression at 4 hours in osteoblastic UAMS‐32 cells^35^ and osteocytic MLO‐Y4 cells,^36^ and was associated with increased CHOP mRNA (Figure 1C,D). In a separate study, Tm also increased the amount of RANKL protein, as determined in extracts of calvaria‐derived osteoblastic cells (Figure 1E).

**Figure 2:**
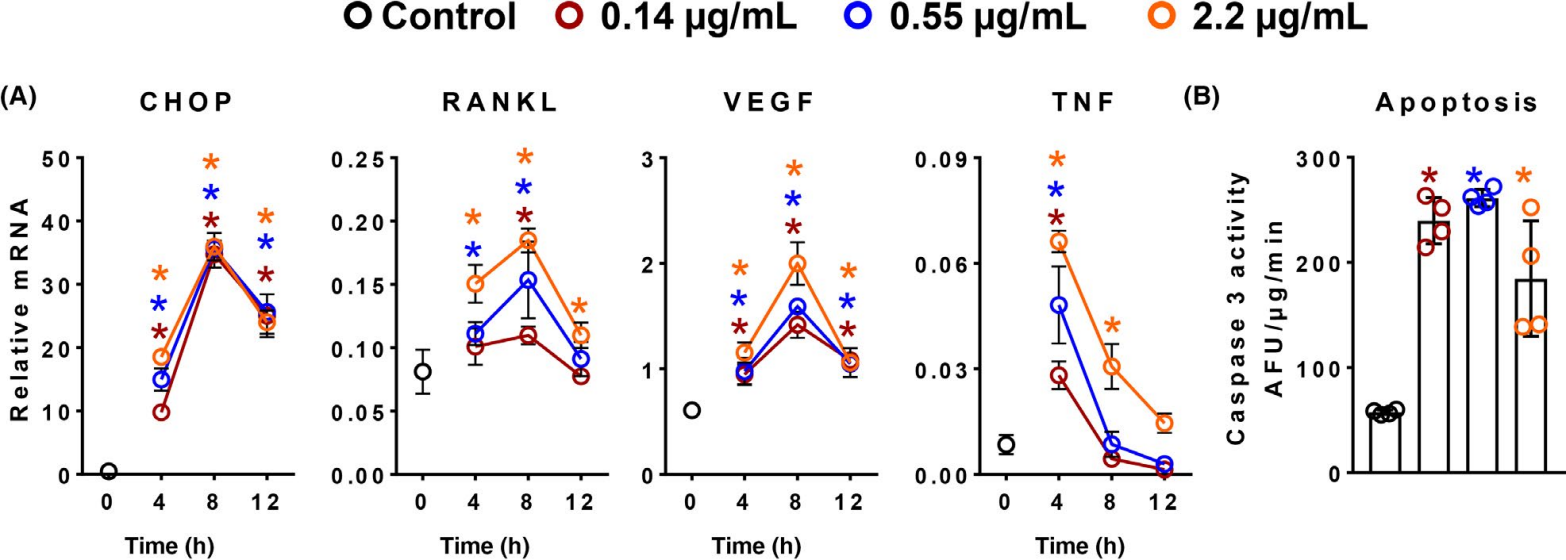
Tm‐induced increase in cytokine expression is dose dependent and transient. Gene expression as determined by qRT‐PCR in calvaria‐derived osteoblastic cells treated with Tm (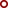 0.14 µg/mL, 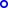 0.55 µg/mL and 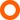 2.2 µg/mL) for indicated times (n = 4/group). Basal gene expression of untreated cells served as control (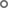). (B) Caspase‐3 activity in calvaria‐derived osteoblastic cells treated with Tm as described in panel A for 12 hours. Data shown are the mean and SD *P < .05 vs control, analyzed by one‐way ANOVA; the color of the asterix denotes statistical significance at the dose tested

We next examined the kinetics of Tm‐induced cytokine expression in calvaria‐derived osteoblastic cells. Tm induced the UPR, as determined by CHOP expression, in a dose‐ and time‐dependent fashion (Figure 2A). The elevation in this marker of UPR was coincident with increased expression of RANKL, VEGF, and TNF. The increase in expression of CHOP and the cytokines was transient, peaking at 4‐8 hours and returning toward baseline by 12 hours, regardless of dose. We also noted that Tm increased apoptosis in these cultures as measured by caspase‐3 activity after 12 hours, (Figure 2B). The latter finding is consistent with prior evidence that cytokine production and cell death are often consequences of prolonged UPR, especially with agents like Tm.^23^, ^24^, ^25^

**Figure 3:**
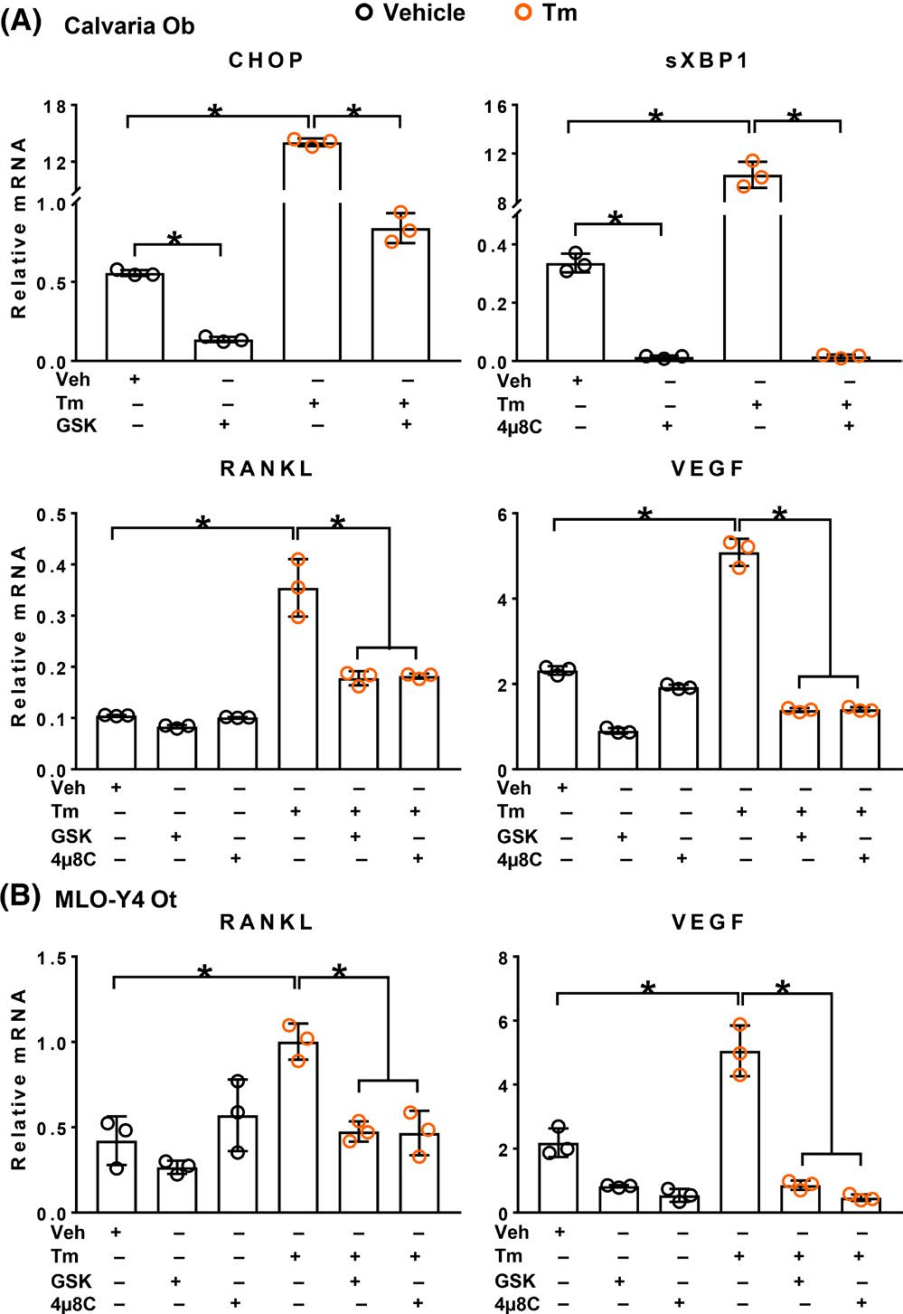
Tm‐induced increase in cytokine expression is blunted by inhibitors of Perk or Ire1α activity. Gene expression as determined by qRT‐PCR in (A) calvaria‐derived osteoblastic cells (Calvaria Ob) and (B) MLO‐Y4 cells (MLO‐Y4 Ot) that were pretreated with either 1 µmol/L GSK2606414 (GSK, PERK inhibitor) or 100 µmol/L 4µ8C (Ire1α inhibitor) for 1 hour, followed by addition of either vehicle (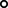, 0.1% DMSO) or 2.2 µg/mL Tm (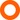, in 0.1% DMSO) for 4 hours. Data shown are the mean and SD *P < .05 analyzed by one‐way ANOVA

To further interrogate the involvement of the UPR in Tm‐stimulated RANKL expression, we used GSK26406414 (GSK) to inhibit PERK activity, and 4µ8C to inhibit IRE1a activity, at concentrations shown to be effective in previous studies.^48^, ^49^ As expected, both these inhibitors suppressed basal levels of CHOP and sXBP1 mRNA, respectively, and attenuated Tm‐induced increase of these UPR target genes in calvaria‐derived osteoblastic cells. More important, both compounds attenuated the Tm‐induced increase in expression of RANKL, well as VEGF, in calvaria‐derived osteoblastic cells (Figure 3A). Similar results were obtained with MLO‐Y4 cells (Figure 3B). These findings provide evidence that Tm‐induced increase in RANKL is linked to the increase in UPR and are unlikely due to toxic side effects of Tm.

### 3.2 Compromising ERAD induces UPR and stimulates RANKL expression in osteoblasts

**Figure 4:**
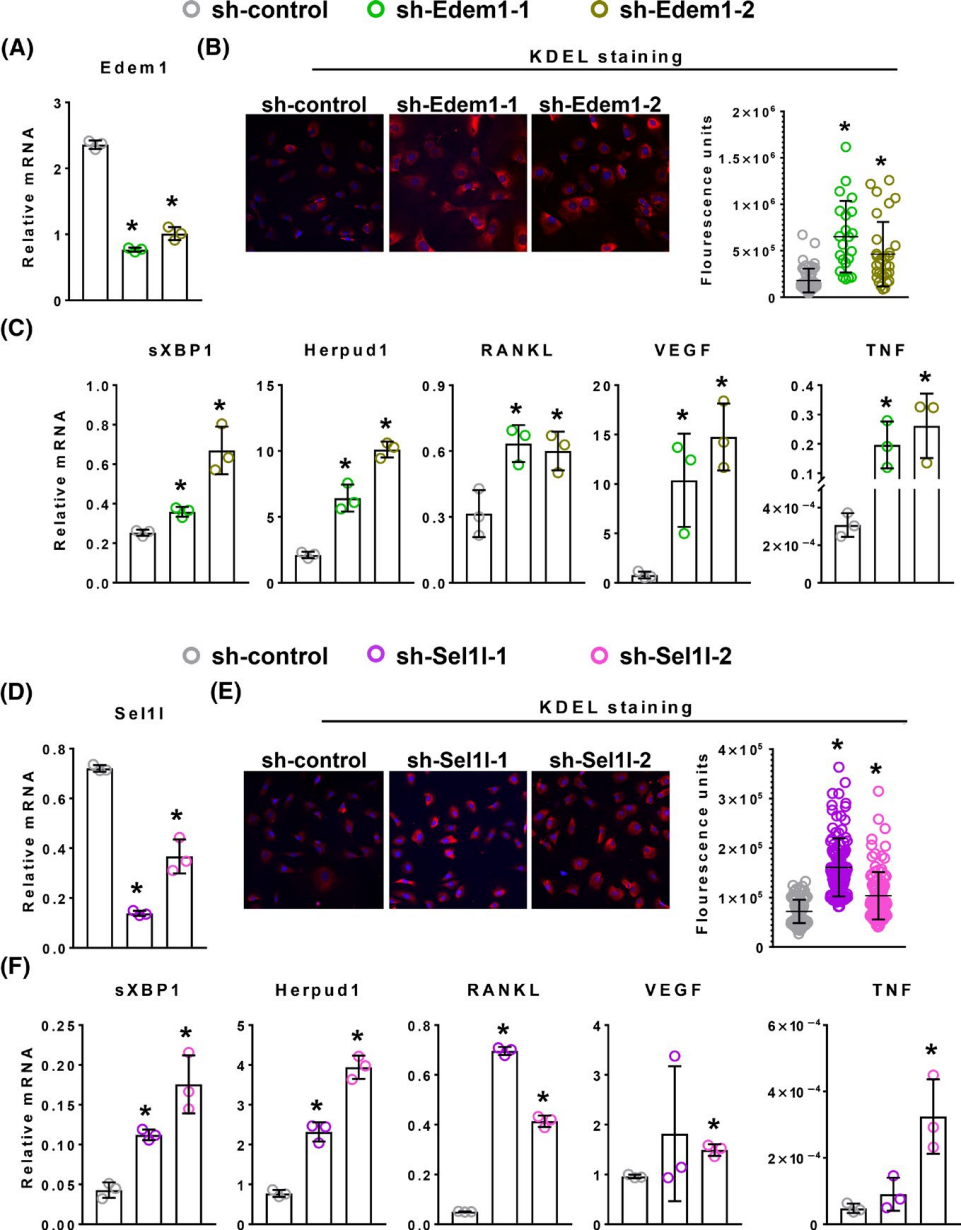
Silencing components of ERAD increases protein retention in the ER, induces UPR, and RANKL expression. Calvarial osteoblasts were infected with using a lentivirus expressing a shRNA directed against (A‐C) Edem1 (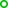 sh‐Edem1‐1 or 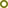 sh‐Edem1‐2) or (D‐F) Sel1l (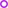 sh‐Sel1l‐1 or 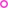 sh‐Sel1l‐2). A nontargeted shRNA served as control (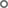 sh‐control). (A, C, D, F) mRNA levels for indicated genes was determined by qRT‐PCR (n = 3/group). (B, E) Proteins in the ER were visualized and quantified (n = 50‐75 cells/group) by confocal microscopy after immunostaining for the ER‐specific peptide KDEL. Nuclei stained with DAPI. Data shown are the mean and SD with individual data points. *P < .05 vs sh‐control by one‐way ANOVA

As a second and independent means of probing the relationship of ER stress and RANKL production, we suppressed expression of two proteins involved in ERAD, specifically Edem1 and Sel1l.^50^, ^51^, ^52^ Calvaria‐derived osteoblastic cells infected with lentivirus expressing either of two short hairpin (sh) RNAs directed against Edem1 (sh‐Edem1‐1 or sh‐Edem1‐2) exhibited the expected reduction in Edem1 mRNA as compared to cells transduced with nontargeted shRNA control (Figure 4A). Importantly, silencing of Edem1 also increased proteins within the ER as indicated by the abundance of KDEL‐containing proteins (Figure 4B). Edem‐1 silencing also induced UPR as measured by increased expression of sXBP1 and Herpud1 (Figure 4C). More importantly, silencing Edem1 also increased the expression of RANKL, VEGF and TNF (Figure 4C). Osteoblastic cells transduced with shRNA directed against Sel1l also increased proteins in the ER and caused an increase in UPR, as well as expression of RANKL, VEGF, and TNF (Figure 4D‐F).

### 3.3 Excess UPR in calvarial organ cultures stimulates bone resorption

**Figure 5:**
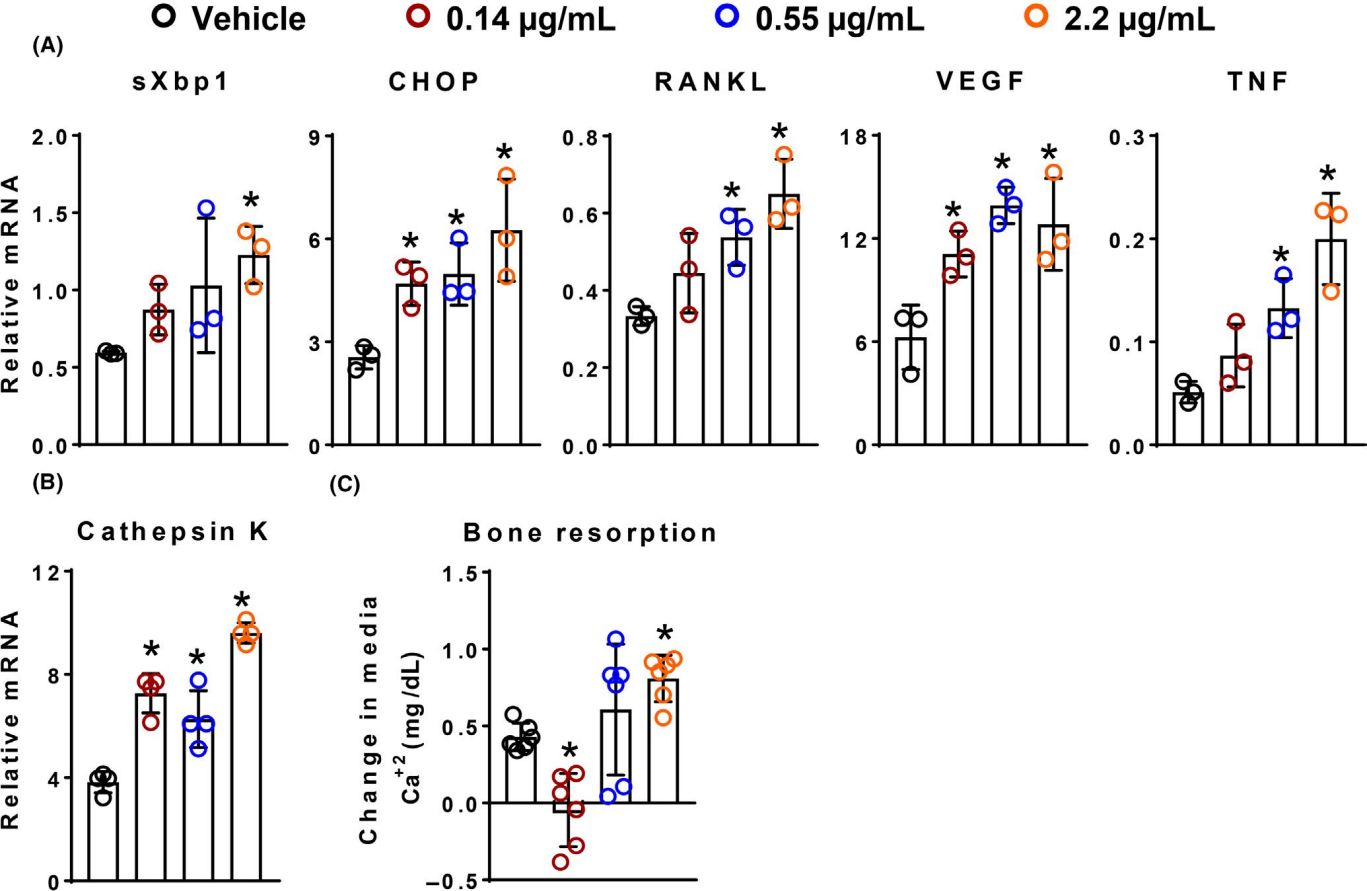
Tm‐induced UPR stimulates expression of RANKL and bone resorption in cultured neonatal murine calvaria. (A) mRNA levels for indicated genes as determined by qRT‐PCR in calvaria treated with either vehicle (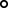 0.1% DMSO) or Tm (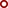 0.14 µg/mL, 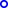 0.55 µg/mL and 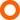 2.2 µg/mL) for 4 hours (n = 3/group). (B) Cathepsin K expression determined by qRT‐PCR in after either vehicle or Tm treatment as in panel A for 4h/ day for 4 days. (C) Bone resorption as determined by the change in medium calcium from the organ cultures described in B. n = 4‐6/group. Data represented as mean and SD with individual data points. *P < .05 vs vehicle by one‐way ANOVA

We next sought to determine whether UPR‐induced RANKL leads to an increase in osteoclasts and bone resorption. To do this, we used organ cultures of intact neonatal murine calvaria.^38^ Figure 2 shows that the Tm‐induced increase in RANKL, and other cytokines was transient, peaking at 4‐8 hours, and that 12 hours of exposure increased apoptosis. Therefore, the calvaria was treated with daily 4 hours pulses of Tm to activate the UPR while minimizing apoptosis. A single 4 hours exposure to Tm increased the UPR, as well as the expression of RANKL, VEGF, and TNF at the highest dose tested (2.2 µg/mL). At lower concentrations, the magnitude of these responses was lower and more variable (Figure 5A). Four daily pulses of Tm increased the expression of the osteoclast marker cathepsin K mRNA at each of the three doses tested (Figure 5B). Tm also increased the release of calcium into the media at the highest dose used, indicating stimulation of bone resorption (Figure 5C).^53^ Paradoxically the lowest dose of Tm suppressed resorption in this study. In three additional experiments (data not shown), 4 hours treatment of 2.2 µg/mL Tm consistently elevated RANKL, as well as cathepsin K expression and bone resportion (measured after four daily 4 hours pulses).

**Figure 6:**
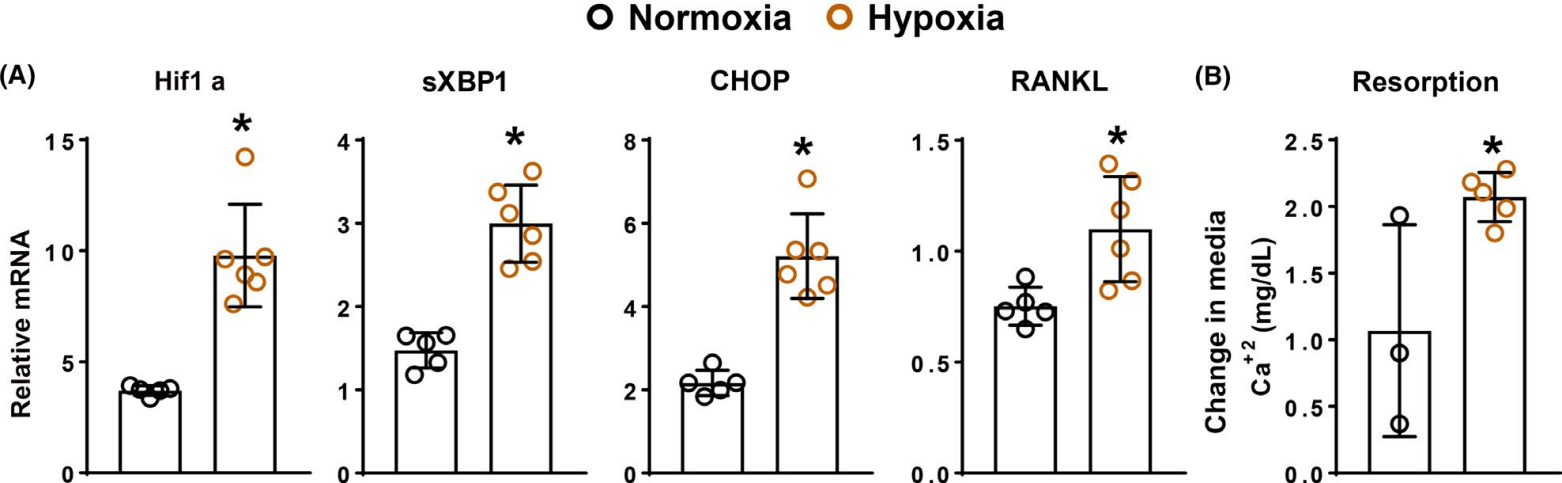
Hypoxia‐induced UPR stimulates RANKL and resorption in cultured neonatal murine calvaria. (A) mRNA levels for indicated genes as determined by qRT‐PCR in calvaria maintained in media that was previously acclimatized to either 20% (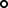 normoxia)or 2% (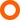 hypoxia) oxygen for 6 hours. (n = 5‐6/group) (B) Bone resorption as determined by the change in calcium in media from organ cultures cultured in the presence of either 20% or 2% oxygen for 6 hours per day for 4 days (n = 3‐5/group). Data represented as mean and SD with individual data points. *P < .05 vs normoxia by Student's *t*‐test

We also examined whether hypoxia, a physiologic inducer of ER stress,^54^, ^55^ can increase RANKL and bone resorption. Six hours of culture under hypoxic conditions (2% O_2_) was sufficient to increase expression of hypoxia inducible factor 1a (Hif1a) in calvaria organ cultures, establishing the potency of this regimen (Figure 6A). This was accompanied by increased UPR as indicated by elevated expression of sXBP1 and CHOP as well as RANKL. Importantly, 4 daily 6‐h bouts of hypoxia increased calcium released into the media (Figure 6B). Collectively these results indicate that pharmacologic or physiologic induction of the UPR in bone organ cultures stimulates RANKL expression leading to increased bone resorption.

### 3.4 Tm‐induced UPR stimulates RANKL expression and bone resorption in mice

**Figure 7:**
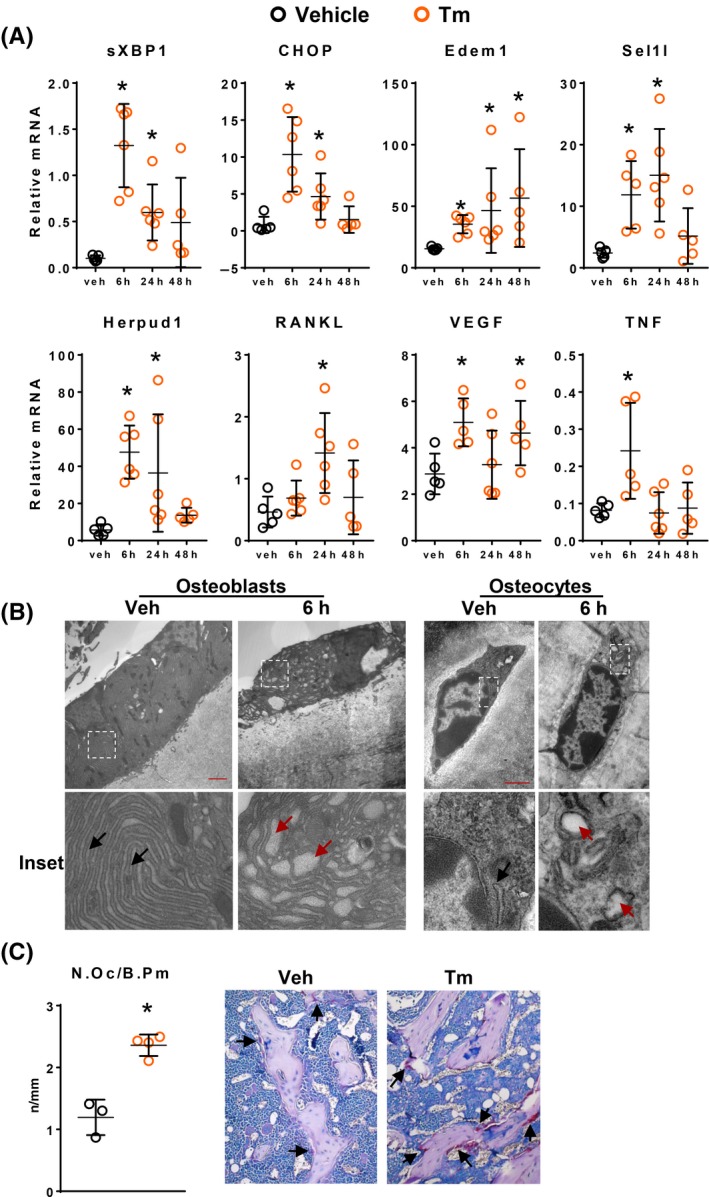
Tm administration to mice‐induced ER stress in osteoblasts and osteocytes, increased RANKL mRNA as well as osteoclast number. (A) mRNA levels for indicated genes as determined by qRT‐PCR in extracts of tibial cortical bone shafts obtained from 10‐week‐old male C57BL/6J mice injected with 5 µg/g body weight Tm (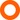) and euthanized at indicated times. Bone from mice injected with 0.5 µL/g body weight DMSO (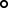, vehicle) and euthanized immediately served as controls (n = 5‐6/group). (B) Representative transmission electron microscope images of osteoblasts and osteocytes in tibial cortical bone from mice euthanized 6 hours after injection of vehicle or Tm in the experiment described in A. Scale bar: 2 µm for osteoblasts and 1 µm for osteocytes. Inset panels show higher power images of the boxed area. The black and red arrows indicate normal and dilated ER, respectively. (C) Osteoclast number per mm cancellous bone surface (N. Oc/B. Pm) in paraffin sections of femora obtained from mice injected with either DMSO (vehicle) or Tm (0.3 µg/g body weight) at 7 and 3 days before euthanasia (n = 3‐4/group). Representative images of TRAPase‐stained paraffin sections are shown on the right. Arrows indicate TRAP + osteoclasts. Data represented as dot plots with mean and SD *P < .05 vs vehicle by (A) one‐way ANOVA or (C) Student's *t*‐test

Finally, we examined whether elevation of UPR in vivo stimulates RANKL expression and osteoclastogenesis in vivo. In these studies, Tm was administered to 2‐3 month old male C57Bl6J mice at 5 µg/g, which has previously been shown to dramatically increase ER stress in vivo.^56^ Six hours after administration, Tm increased mRNA for sXBP1, CHOP, Edem1, and Sel1l in preparations of femoral cortical bone, followed by a decline towards basal levels by 24‐48 hours. RANKL mRNA levels were also elevated, but only at 24 hours, whereas VEGF and TNF expression was increased at 6 hours (Figure 7A). This cortical bone preparation mainly consists of osteocytes, but endosteal osteoblasts, lining cells, and osteoclasts are also present.^57^, ^58^ To establish that Tm indeed caused ER stress in osteoblasts and osteocytes in this study, we examined cortical bone of the tibia from the same animal using transmission electron microscopy. Six hours after Tm injection, both cell types exhibited dilated ER (Figure 7B), a well‐established morphologic indicator of ER stress.^46^

In a separate study, we assessed the impact of elevated UPR on osteoclast number. RANKL‐induced osteoclastogenesis requires several days,^30^ and Tm‐induced UPR and cytokine expression in bone is transient (Figure 7A). Therefore, we administered Tm to 4‐month‐old female C57BL/6J mice twice over a 4 d period at 0.3 µg/g, a dose that induces ER stress in vivo without causing lethality,^32^ and then euthanized the animals 3 days after the last injection. Mice injected with Tm exhibited a reduction in body weight (veh, 5.3% ± 1.5%; Tm, −11.7% ± 7.5%, *P* = .01). Histomorphometric evaluation of femoral cancellous bone revealed that Tm increased the number of TRAP positive osteoclasts (Figure 7C). However, expression of markers of UPR activation, and of RANKL mRNA, was unaffected as measured at the end of the study (data not shown), most likely due to the transient nature of these responses noted as in Figure 7A.

## 4 DISCUSSION

Our findings demonstrate that RANKL is one of the cytokines produced in response to elevated UPR signaling caused by Tm, suppression of ERAD, or hypoxia in cultures of osteoblasts, osteocytes, or calvaria organ cultures. In the latter, Tm or hypoxia also increased bone resorption; and administration of Tm to mice increased RANKL expression in bone, and increased osteoclast number in trabecular bone. Besides RANKL, increased UPR signaling stimulated the expression of VEGF and TNF, consistent with previous studies in neuroblastoma cells and macrophages.^59^, ^60^ Furthermore, inhibitors of either Perk or Ire1a activity blunted the Tm‐induced increase in RANKL mRNA in cultured cells, thus linking the UPR with RANKL transcription. A previous study demonstrating reduced RANKL expression in bones of mice with germline deletion of Perk further supports this conclusion.^61^ It is likely that UPR affects multiple cytokines in osteoblasts and osteocytes in addition to those described in this report. Besides VEGF and TNF, the UPR could have affected other locally produced cytokines that have been shown to influence RANKL expression such as IL‐1, IL‐6, IL‐11, oncostatin M and leukemia inhibitory factor.^14^, ^15^ Additional studies will be required to ascertain the cytokines profile in response to elevated UPR in bone cells and better understand mechanism(s) – distinct or common – underlying their regulation and the interdependence of the affected cytokines in promoting bone resorption.

The increase in RANKL in calvaria‐derived osteoblasts following Tm exposure was transient in contrast to the sustained elevation caused by obstruction of ERAD via silencing of Edem1 or Sel1l. This difference might reflect the more intense UPR caused by Tm as compared to the ER stress caused by inhibition of ERAD. Indeed the UPR is much lower in cells with reduced ERAD than in Tm treated cells, as reflected by sXBP1 expression in the studies reported herein. This contention is supported by other studies showing that Sel1l deficiency causes a moderate induction of UPR in neurons, adipocytes and hepatocytes in vivo along with increased production of fibroblast growth factor 21 in the liver.^62^, ^63^, ^64^, ^65^

We found that stimulation of UPR by Tm or hypoxia‐stimulated osteoclastogenesis and bone resorption in cultures of neonatal calvaria, and that repeated administration of Tm increased osteoclast number in trabecular bone of adult mice. These responses are most likely due to increased expression of RANKL by osteoblasts and osteocytes, perhaps in conjunction with other cytokines stimulated by the UPR. Indeed, we consistently observed a UPR‐induced increase in RANKL expression in osteoblast and osteocyte cell lines, cultured neonatal calvaria, calvaria‐derived osteoblasts, and marrow‐free cortical bone preparations. It was previously reported that an increase in UPR is involved in RANKL‐stimulated osteoclastogenesis.^66^ Thus, Tm‐induced resorption observed in bone organ cultures and Tm‐induced increase in osteoclast number in mice could be due, in part, to increased UPR in these cells. Elevated UPR has been associated with increased osteoclasts and bone loss in a rats with osteonecrosis of the femoral head,^67^ mice with periodontitis induced by in *P gingivalis*,^68^ as well as mouse models of hind limb unloading and particle‐induced osteolysis.^69^, ^70^ Notably, the latter two models also reported an increase in RANKL in whole bone extracts. Cytokines like TNF and γ‐interferon have recently been shown to increase the UPR via production of ROS and/or depletion of calcium within the ER. Thus, these cytokines may activate a positive feedback loop that exacerbates ER stress.^71^, ^72^ The findings of this report link elevated UPR with increased expression of RANKL, and other cytokines like TNF and VEGF. It is therefore possible that elevated UPR may be involved in the increased RANKL and osteoclast number leading to bone loss in a variety of disorders, particularly those associated with inflammatory conditions like arthritis, orthopedic implant‐associated osteolysis, and periodontitis.^6^, ^10^ In summary, our findings together with these earlier reports set the stage for additional studies to investigate the role of the UPR in pathologic bone loss.

## CONFLICT OF INTEREST

The authors state that they have no conflicts of interest.

## AUTHOR CONTRIBUTIONS

S. Iyer and R. L. Jilka designed research; S. Iyer, C. Melendez‐Suchi, and L. Han performed the research; S. Iyer, M. Almeida, and R. L. Jilka analyzed data; S. Iyer and R. L. Jilka wrote the manuscript and; G. Baldini and M. Almeida assisted in manuscript revision.
